# Self-organization of early vocal development in infants and machines: the role of intrinsic motivation

**DOI:** 10.3389/fpsyg.2013.01006

**Published:** 2014-01-16

**Authors:** Clément Moulin-Frier, Sao M. Nguyen, Pierre-Yves Oudeyer

**Affiliations:** Flowers Team, Institut National de Recherche en Informatique et en Automatique / ENSTA-ParistechBordeaux, France

**Keywords:** vocal development, intrinsic motivation, curiosity-driven learning, imitation, self-organization, interactive learning, goal babbling

## Abstract

We bridge the gap between two issues in infant development: vocal development and intrinsic motivation. We propose and experimentally test the hypothesis that general mechanisms of intrinsically motivated spontaneous exploration, also called curiosity-driven learning, can self-organize developmental stages during early vocal learning. We introduce a computational model of intrinsically motivated vocal exploration, which allows the learner to autonomously structure its own vocal experiments, and thus its own learning schedule, through a drive to maximize competence progress. This model relies on a physical model of the vocal tract, the auditory system and the agent's motor control as well as vocalizations of social peers. We present computational experiments that show how such a mechanism can explain the adaptive transition from vocal self-exploration with little influence from the speech environment, to a later stage where vocal exploration becomes influenced by vocalizations of peers. Within the initial self-exploration phase, we show that a sequence of vocal production stages self-organizes, and shares properties with data from infant developmental psychology: the vocal learner first discovers how to control phonation, then focuses on vocal variations of unarticulated sounds, and finally automatically discovers and focuses on babbling with articulated proto-syllables. As the vocal learner becomes more proficient at producing complex sounds, imitating vocalizations of peers starts to provide high learning progress explaining an automatic shift from self-exploration to vocal imitation.

## 1. Introduction

### 1.1. Vocal development and intrinsic motivation

Early on, babies seem to explore vocalizations as if it was a game in itself, as reported by Oller (2000) who cites two studies from the nineteenth century:

“[At] 3 months were heard, for the first time, the loud and high crowing sounds, uttered by the child sponteaneously, […] the child seemed to take pleasure in making sounds.” (Sigismund, 1971)

“[He] first made the sound *mm* spontaneously by blowing noisily with closed lips. This amused [him] and was a discovery for [him].”^1^ (Taine, 1971)

Such play with his vocal tract, where the baby discovers the sounds he can make, echoes other forms of body play, such as exploration of arm movements or how he can touch, grasp, mouth or throw objects. The concept of *intrinsic motivation* has been proposed in psychology to account for such spontaneous exploration (Berlyne, 1954; Deci and Ryan, 1985; Csikszentmihalyi, 1997; Ryan and Deci, 2000; Gottlieb et al., 2013):

“Intrinsic motivation is defined as the doing of an activity for its inherent satisfaction rather than for some separable consequence. When intrinsically motivated, a person is moved to act for the fun or challenge entailed rather than because of external products, pressures or reward.” (Ryan and Deci, 2000)

Intrinsic motivation refers to a mechanism pushing individuals to select and engage in activities for their own sake because they are inherently interesting (in opposition to *extrinsic motivation*, which refers to doing something because it leads to a separable outcome). A key idea of recent approaches to intrinsic motivation is that *learning progress* in sensorimotor activities can generate intrinsic rewards in and for itself, and drive such spontaneous exploration (Gottlieb et al., 2013). Learning progress refers to the infant's improvement of his predictions or control over activity they practice, which can also be described as reduction of uncertainty (Friston et al., 2012).

Although spontaneous vocal exploration is an identified phenomenon, occurring in the early stages of infant development, the specific mechanisms of such exploration and the role of intrinsic motivation for the *structuration* of early vocal development has not received much attention so far to our knowledge. We propose that mechanisms of intrinsically motivated spontaneous exploration, which we also refer to as curiosity-driven learning, play an important role in speech acquisition, by driving the infant to follow a self-organized developmental sequence which will allow him to progressively learn to control his vocal tract. This is to our knowledge a largely unexplored hypothesis. The goal of this article is to formalize in detail this hypothesis and study general properties of such mechanisms in computer experiments.

Several computational models of speech development, where speech acquisition is organized along a developmental pathway, have been elaborated so far. They have shown how such stage-like organization can ease the acquisition of complex realistic speech skills.

The DIVA model (Guenther et al., 1998; Guenther, 2006), as well as Kröger's model (Kröger et al., 2009), propose architectures partly inspired by neurolinguistics. They involve two learning phases. The first one is analogous to infant babbling and corresponds to semi-random articulator movements producing auditory and somatosensory feedbacks. This is used to tune the correspondences between representation maps within a neural network. In the second phase, the vocal learner is presented with external speech sounds analogous to an ambient language and learns how to produce them adequately. The Elija model (Howard and Messum, 2011) also distinguishes several learning phases. In the first phase of exploration, the agent is driven by a reward function, including intrinsic rewards such as sound salience and diversity, as well as articulatory effort. Various parameterizations of this reward function allows the model to produce vocalizations in line with Oller's vocal developmental stages of infants. In a subsequent phase, the sounds produced by the model attract the attention of a caregiver, providing an external reinforcement signal. Other models also use a reinforcement signal, either from human listeners [social reinforcement (Warlaumont, 2012, 2013b)] or based on sound saliency [intrinsic reinforcement (Warlaumont, 2013a)], and show how this can influence a spiking neural network to produce canonical syllables. Such computational models of speech acquisition pre-determine the global ordering and timing of learning experiences, which amounts to preprograming the developmental sequence. Understanding how a vocal developmental sequence can be formed is still a major mystery to solve, and this article attempts a first step in this direction.

We build on recent models of skill learning in other modalities (e.g., locomotion or object manipulation), where it was shown that mechanisms of intrinsically motivated learning can self-organize developmental pathways, adaptively guiding exploration and learning in high-dimensional sensorimotor spaces, involving highly redundant and non-linear mappings (Oudeyer et al., 2007; Baranes and Oudeyer, 2013; Gottlieb et al., 2013; Oudeyer et al., 2013). Such models concretely formalize concepts of intrinsic motivation described in the psychology literature into algorithmic architectures that can be experimented in computers and robots (Schmidhuber, 1991; Barto et al., 2004; Oudeyer and Kaplan, 2007; Baldassarre, 2011). Detailed discussions of the engineering aspects of such intrinsic motivation mechanisms, casted in the statistical framework of active learning, have been recently published and showed their algorithmic efficiency to learn sensorimotor coordination skills in redundant non-linear high-dimensional mappings (Baldassarre and Mirolli, 2013; Baranes and Oudeyer, 2013; Srivastava et al., 2013).

Indeed, transposed in curiosity-driven learning machines (Schmidhuber, 1991; Barto et al., 2004; Schembri et al., 2007; Hart, 2009; Merrick and Maher, 2009; Schmidhuber, 2010; Stout and Barto, 2010) and robots (Oudeyer et al., 2007; Baranes and Oudeyer, 2013), these developmental mechanisms have been shown to yield highly efficient learning of inverse models in high-dimensional redundant sensorimotor spaces (Baranes and Oudeyer, 2010, 2013). These spaces share many mathematical properties with vocal spaces. Efficient versions of such mechanisms are based on the active choice of learning experiments that maximize learning *progress*, e.g., improvement of predictions or of competences to reach goals (Schmidhuber, 1991; Oudeyer and Kaplan, 2007; Oudeyer et al., 2007; Baranes and Oudeyer, 2013; Srivastava et al., 2013). Such learning experiments are called “progress niches” (Oudeyer et al., 2007).

Yet, beyond pure considerations of learning efficiency, exploration driven by intrinsic rewards measuring learning progress was also shown to self-organize structured developmental pathways, both behaviorally and cognitively. Indeed, such mechanisms automatically drive the system to explore and learn first easy skills, and then progressively explore skills of increasing complexity (Oudeyer et al., 2007). They have been shown to generate automatically behavioral and cognitive developmental structures and have been analyzed in relation to their similarities with infant development (Oudeyer and Kaplan, 2006; Kaplan and Oudeyer, 2007a; Oudeyer et al., 2007; Moulin-Frier and Oudeyer, 2012). For example, in the Playground Experiment, a curiosity-driven learning robot was shown to self-organize its own learning experiences into a sequence of behavioral and cognitive stages where it spontaneously acquired various affordances and skills of increasing complexity (Oudeyer et al., 2007). It was also shown how it could discover and focus on elementary vocal interaction with a peer as a spontaneous consequence of its general drive to explore situations where it can improve its predictions (Oudeyer and Kaplan, 2006). Focusing on vocal interactions was thus explained as a special case of focusing on an activity that provides learning progress (i.e., a particular progress niche). This therefore allowed to generate some novel hypotheses to explain infant development, from the behavioral (Oudeyer and Kaplan, 2006), cognitive (Kaplan and Oudeyer, 2007a), or brain circuitry (Kaplan and Oudeyer, 2007b) perspectives [see Gottlieb et al. (2013) for a review on these novel perspectives]. Intrinsically motivated spontaneous learning has also been combined with mechanisms of imitation learning within the SGIM-ACTS architecture, as detailed in Nguyen and Oudeyer (2012). In this model, formulated within the framework of strategic learning (Lopes and Oudeyer, 2012), a hierarchical active learning architecture allows an interactive learning agent to choose by itself when to explore autonomously, and when, what and who to imitate, based on measures of competence progress.

Although intrinsic motivation and socially guided learning have already been considered in computational models specifically studying speech acquisition, to our knowledge, they have so far been considered as two distinct learning phases with a hard-coded switch between them (e.g., Guenther et al., 1998; Guenther, 2006; Kröger et al., 2009; Howard and Messum, 2011). In other words, the existence of distinct developmental stages was presupposed in these models. In contrast, these distinct learning phases emerge from the Playground Experiment, even though only a simplistic vocal system was considered (only pitch and duration were controlled, and no physical model of the vocal tract was used; modeling of speech acquisition *per se* was not the focus of this study).

Our main contribution in this paper is to show how mechanisms of intrinsically motivated exploration applied on a realistic articulatory-auditory system self-organizes autonomously into coherent *vocal* developmental sequences. This follows the approach of our previous works (Moulin-Frier and Oudeyer, 2012, 2013a,b), which were preliminary studies limited to vowel production and focusing only on autonomous learning, i.e., without considering a surrounding ambient language.

In such a conceptual framework, developmental structures are neither learnt from “tabula rasa” nor a pre-determined result of an innate “program”: they self-organize out of the dynamic interaction between constrained cognitive mechanisms (including curiosity, learning, and abstraction), the morphological properties of the body, and the physical and social environment which itself is constrained and ordered by the developmental level of the organism (Thelen and Smith, 1996; Oudeyer et al., 2007). Thus, the approach we take can be viewed as an instantiation of the concept of epigenesis, in the sense proposed by (Gottlieb, 1991).

The study of such a dynamical systems approach, where curiosity-driven learning is an important force, can take ample advantage of computer modeling as a research tool. Here in particular, it can help to understand better the dynamics underlying early vocal development, and in particular understand what are the mechanisms which generate the developmental sequence(s) in vocal productions and capabilities observed in infants. In particular, it can help to understand what is the precise role of intrinsic motivation.

In the next sections of this introduction, we summarize properties of vocal development during the first year and describe the general principles of the computational model we study in this article.

### 1.2. Development of vocalizations

**Figure 1:**
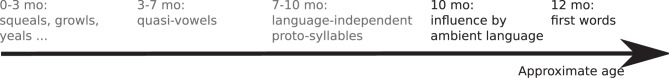
**The first year of infant vocal development**.

Despite inter-individual variations in infant vocal development (e.g., Vihman et al., 1986), strong regularities in the global structuration of vocal development are identified (Oller, 2000; Kuhl, 2004). In this article, we adopt the view from Oller (2000) as well as Kuhl (2004). Figure 1 schematizes this vocal development during the first year of infant. It can be summarized as follows. First, until the age of approximately 3 months, an infant produces non-speech sounds like squeals, growls and yeals. During this period, he seems to learn to control infrastructural speech properties, e.g., phonation and primitive articulation (Oller, 2000). Then, from 3 to 7 months, he begins to produce vowel-like sounds (or quasi-vowels) while he probably learns to control his vocal tract resonances. At 7 months, canonical babbling emerges where well-timed sequences of proto-syllables are mastered. But it is only around the age of 10 months that infant vocal productions become more influenced by the ambient language, leading to first word productions around 1 year of age.

Two features of this developmental sketch are particularly salient.

- Infants seem to first play with their vocal tracts in a relatively language-independent way, and then are progressively influenced by the ambient speech sounds.
- In the initial phase, when sounds produced by their peers influence little their vocalizations, infants seem to learn skills of increasing complexity: normal phonation, then quasi-vowels and finally proto-syllables. According to Oller (2000), such a sequence displays a so-called natural, or logical hierarchy. For example, it is impossible to master quasi-vowel production without previously mastering normal phonation.

### 1.3. A computational model of vocal development

To articulate hypotheses about the possible roles of intrinsic motivation in the first year of vocal development, we build here a computational model of an intrinsically motivated vocalizing agent, in contact with vocalizations of peers. In the model, an individual speech learner has the following characteristics, described in detail in next sections:

- It embeds a realistic model of a human vocal tract: the articulatory synthesizer used in the DIVA model (Guenther et al., 2006). This model provides the way to produce sequences of vocal commands and to compute corresponding sequences of acoustic features, both in multi-dimensional continuous domains.
- It embeds a dynamical model for producing motions of the vocal tract, based on a an over-damped spring-mass model. This model describes dynamical aspects such as co-articulation in sequences of vocal targets.
- It is able to iteratively learn a probabilistic sensorimotor model of the articulatory-auditory relationships according to its own experience with the vocal tract model. Because the sensorimotor learning is iterative during the life time of the agent, it will first be inefficient at using this model for control, and then progresses by learning from its own experience.
- It is equipped with an intrinsically motivated exploration mechanism, which allows it to generate and select its own auditory goal sequences. Such mechanism includes a capability to empirically measure its own competence progress to reach sequences of goals. Then, an action selection system stochastically self-selects target goals that maximize competence progress.
- It is able to hear sounds of a simulated ambient language, and its intrinsic motivation system is also used to decide whether to self-explore self-generated auditory goals, or to try to emulate adult sounds. This choice is also based on a measure of competence progress for each strategy.

Then, we present experiments allowing us to study how the developmental structuration of early vocal exploration could be self-organized in an intrinsically motivated speech learner, under the influence of sounds in the environment and constrained by the physical properties of the sensorimotor system.

In a first series of experiments, we consider a speech learner who is not exposed to external speech sounds. This allows the study of the role of intrinsic motivation independently of any social influence. We show how a cognitive architecture for intrinsically motivated autonomous exploration (SAGG-RIAC; Baranes and Oudeyer, 2013; Moulin-Frier and Oudeyer, 2013a), applied to learning to control an articulatory synthesizer (i.e., a vocal tract model able to produce speech sounds from articulatory configurations), can self-organize coherent vocal developmental sequences. This work extends preliminary studies (Moulin-Frier and Oudeyer, 2012, 2013a,b) through the use of a different vocal tract model and a more complex model of motion control dynamics with an overdamped spring-mass dynamical system, providing the agent with a more realistic and powerful mechanism to produce (un)articulated sounds.

In a second series of experiments, the speech learner is exposed to speech sounds from its environment. The cognitive architecture is extended to strategic interactive intrinsically motivated learning (SGIM-ACTS; Nguyen and Oudeyer, 2012), where intrinsic motivation is also used by the learner to decide when to self-explore and when to try to imitate sounds in the environment. In the present study, we suppose that the sounds of the adult are directly imitable (we do not account for the pitch and formant differences between infants and adults for instance). We show that the system first focuses on self-exploration of vocalization. It later on shifts to vocal imitation, which then influences its vocal learning in ways that are specific to the speech environment. Yet, in this paper, we do not study the social interaction aspect of the teacher and, in particular, we do not model the behavior of the adult in response to the learner behavior.

Our aim is to study how important aspects of infant vocal development in the first year of life, described in the previous section, could be explained by the interaction between these building blocks: an intrinsic motivation system, a dynamic motor system associated to morphological and physiological constraints, an imitation system and a system for learning a sensorimotor model out of physical experiments. We will show that competence progress based autonomous exploration is able to provide a unified explanation for both the tendency to produce vocalizations of increasing complexity and the progressive influence of the ambient adult sounds. Imitating adult sounds becomes interesting for the speech learner only when basic speech production principles have been previously mastered. Contrarily to existing models of speech acquisition we described so far, our aim is not to reproduce infant vocalizations in a phonetically detailed manner, but rather to suggest an hypothesis about how a succession of distinct developmental stages can self-organize autonomously. Howard and Messum's model (Howard and Messum, 2011) for example, shows how distinct parameterizations of an intrinsic reward function can enable a vocal agent to discover several type of sounds coherent with observed developmental stages in infants. These parameterizations however, are hard-coded. In contrast, our model is not designed to reproduce precisely infant vocalizations within distinct vocalization stages, but rather to understand how the *transition* from one stage to another can be explained by a drive to maximize the competence progress to reach self-generated or ambient auditory goals. In consequence, the switch from self-generated auditory goals to the imitation of adult sounds is not hard-coded in our model, but emerges as a by-product of the drive to focus on progress niches.

## 2. Model

In this section, we describe the models that we use for the vocal tract and auditory signals. We describe the learning of the internal model of the sensorimotor mapping, and the intrinsic motivation mechanism which allows the learner to decide adaptively which vocalization to experiment at given moments during its development, and whether to do so through self-exploration or through imitation of external sounds.

### 2.1. Sensorimotor system

#### 2.1.1. Vocal tract and auditory system

**Figure 2:**
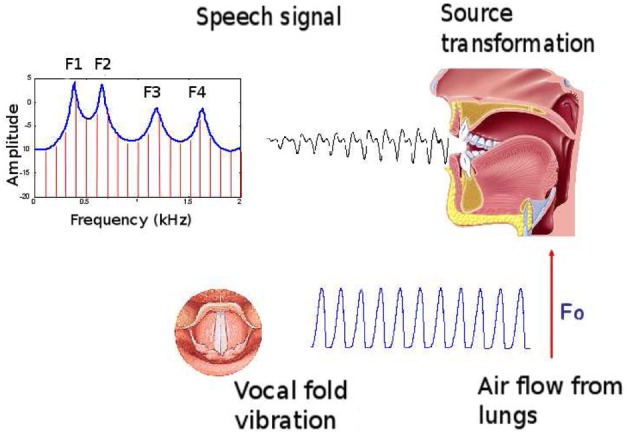
**Speech production general principles**. The vocal fold vibration by the lung air flow provides a source signal: a complex sound wave with fundamental frequency *F*_0_. According to the vocal tract shape, acting as a resonator, the harmonics of the source fundamental frequency are selectively amplified or faded. The local maxima of the resulting spectrum are called the formants, ordered from the lower to the higher frequencies. They belong to the major features of speech perception.

**Figure 3:**
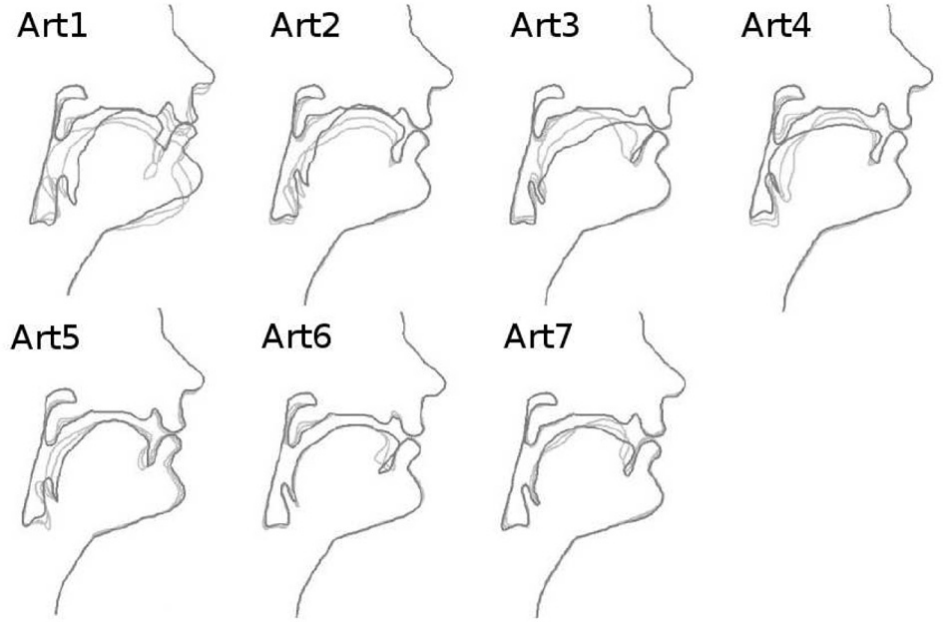
**Articulatory dimensions controlling vocal tract shape (10 dimensions, from left to right and top to bottom), adapted from the documentation of the DIVA source code**. Each subplot shows a sagittal contour of the vocal tract, where we can identify the nose and the lips on the right side. Bold contours correspond to a positive value of the articulatory parameter, the two thin contours are for a null (neutral position) and negative values. These dimensions globally correspond to the dimensions of movements of the human vocal tract articulators. For example, *Art*_1_ mainly controls the jaw height, whereas *Art*_3_ rather controls the tongue front-back position.

Our computational model involves the articulatory synthesizer of the DIVA model described in Guenther et al. (2006)^2^. based on Maeda's model (Maeda, 1989). Without going into technical details, the model corresponds to a computational approximation of the general speech production principles illustrated in Figure 2. The model receives 13 articulatory parameters as input. The first 10 are from a principal component analysis (PCA) performed on sagittal contours of images of the vocal tract of a human speaker, allowing to reconstruct the sagittal contour of the vocal tract from a 10-dimensional vector. The effect of the 10 articulatory parameters from the PCA on the vocal tract shape is displayed Figure 3. In this study, we will only use the 7 first parameters (the effect of the others on the vocal tract shape is negligible), fixing the 3 last in the neutral position (value 0 in the software). Through an area function, associating sections of the vocal tract with their respective area, the model can compute the 3 first formants of the resulted signal if phonation occurs. Phonation is controlled through the 3 last parameters: glottal pressure controlling the intensity of the signal (from quiet to loud), voicing controlling the voice (from voiceless to voiced) and pitch controlling the tone (from low-pitched to high-pitched). It is then able to compute the formants of the signal (among other auditory and somato-sensory features) through the area function. In this study, we only use the glottal pressure and voicing parameters. In addition to the 7 articulatory parameters from the PCA, a vocal command is therefore defined by a 9-dimensional vector. From the vocal command, the synthesizer computes the auditory and somatosensory consequences of the motor command, thus approximating the speech production principles of Figure 2.

On the perception side of our model, we use the first two formants of the signal, *F*1 and *F*2, approximately scaled between −1 and 1. We also define a third parameter *I* which measures the intensity (or phonation level) of the auditory outcome. *I* is supposed to be 0 when the agent perceives no sound, and 1 when it perceives a sound. Technically, *I* = 1 if and only if two conditions are checked: (1) both pressure and voicing parameters are above a fixed threshold (null value) and (2) the vocal tract is not closed (i.e., the area function is positive everywhere). In human speech indeed, the formants are not measurable when phonation is under a certain threshold. We model this by setting that when *I* = 0, the formants do not exist anymore and are set to 0. This drastic simplification is yet arguable in term of realism, but what we want to model here is the fact that no control of the formant values can be learnt when no phonation occurs.

#### 2.1.2. Dynamical properties

Speech production and perception are dynamical processes and the principles of Figure 2 have to be extended with this respect. Humans control their vocal tract by variations in muscle activations during a vocalization, modulating the produced sound in a complex way. Closure or opening movements during a particular vocalization, coupled with variations in phonation level, are able to generate a wide variety of modulated sounds. We thus define a vocalization as a trajectory of the 9 motor parameters over time, lasting 800 ms, from which the articulatory synthesizer is able to compute the corresponding trajectories in the auditory space (i.e., trajectories in the 3-dimensional space of *F*1, *F*2, and *I*). The agent is able to control this trajectory by setting 2 commands for each articulator: one from 0 to 250 ms, the other one from 250 to 800 ms. Then, the motor system is modeled as an overdamped spring-mass system driven by the following second-order dynamical equation:

(1) $$\ddot{x}+2\zeta \omega_{0}\dot{x}+\omega_{0}^{2}(x-m)=0,$$

where *x* is a motor parameter, and *m* is the command for that motor parameter. ζ is set to 1.01, ensuring that the system is overdamped (no oscillation), and ω_0_ to $\frac{2\pi }{0.8}$ (0.8 being the duration of the vocalization in seconds). Thus, the agent's policy for a vocalization is defined by two vectors *m*_1_ and *m*_2_ (one for each command) of 9 real values each (one for each motor parameter). The policy space is 18-dimensional. The first command is applied for the beginning of the vocalization to 250 ms, the second one from 250 to 800 ms.

**Figure 4:**
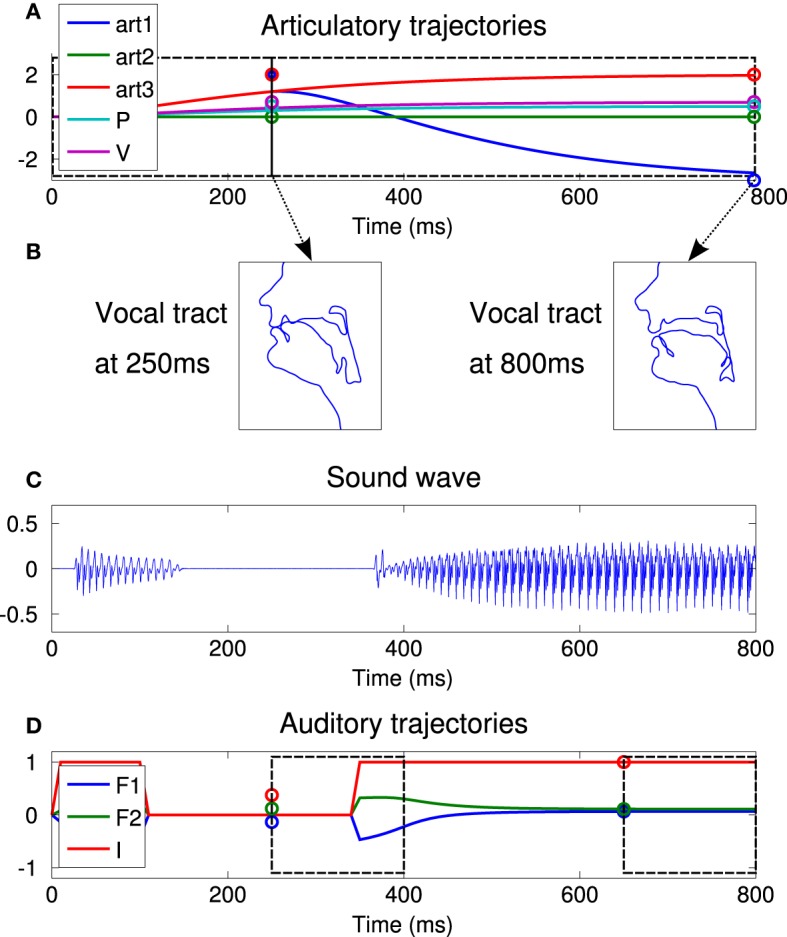
**An illustrative vocalization example**. **(A)** Articularory trajectories of 5 articulators during the 800 ms of the vocalization (4 articulators, from *art*4 to *art*7 are not plotted for the sake of readability but display the same trajectory as *art*2). Circles at 250 and 800 ms represents the values of the first and second commands, respectively, for each trajectory. The first commands are active from 0 to 250 ms and second ones from 250 to 800 ms, as represented by dotted black boxes. The trajectories are computed by the second order dynamical Equation (1), starting in a neutral position (all articulators set to 0). **(B)** Resulting vocal tract shapes at the end of each command, i.e., at 250 and 800 ms. Each subplot displays a sagittal view with the nose and the lips on the left side. The tongue is therefore to the right of the lower lip. **(C)** Sound wave resulting from the vocalization. **(D)** Trajectories of the 3 auditory parameters, the intensity *I* and the two first formants *F*1 and *F*2. Dotted black boxes represent the two perception time windows. The agent perceives the mean value of the auditory parameters in each time window, represented by the circles at 250 and 650 ms.

Figure 4A illustrates the process by showing a typical syllabic vocalization. In this illustrative example, the controlled articulators are the first and third articulators of Figure 3 (roughly controlling the jaw height and the tongue front/back dimensions), as well as pressure and voicing. The two last ones are set to 0.5 and 0.7, respectively, for both commands, to allow phonation to occur. The “jaw parameter” (*art*1 on the figure) is set to 2.0 (jaw closed) for the first command and to −3.0 for the second one (jaw open). We observe that these commands, quite far from the neutral position, are not completely reached by the motor system. This is due to the particular dynamics of the system, defined with ζ and ω_0_ in the dynamical system. For the third articulator (*art*3), the commands are both at 2.0. We observe that, whereas the value 2.0 cannot be achieved completely at 250 ms, it can however be reached before the end of the vocalization.

This motor system implies interaction between the two commands, i.e., a form of co-articulation. Indeed, a given motor configuration may sometimes be harder to reach if it is set as the first command, because time allocated to reach the first command is less than for the second command. Reversely, some movements may be harder to control in the second command because the final articulator positions will depend both on the first and the second commands (e.g., it is harder to reach the value −3.0 for the second command if the first command is set to 2.0, than if the first command is set to −3.0, as seen in the example of Figure 4).

These characteristics are the results of modeling speech production as a damped spring-mass system (Equation 1), which is a common practice in the literature (Markey, 1994; Boersma, 1998; Howard and Messum, 2011).

Figure 4B shows the resulting vocal tract shape at the end of the 2 commands (i.e., at 250 ms and at 800 ms). We observe that the vocal tract is closed at the end of the first command, open at the end of the second one.

Figure 4C shows the resulting sound. We observe that there is no sound during vocal tract closure.

Figure 4D shows the resulting trajectories of auditory parameters. In our experiments, we model the auditory perception of the agent of its own vocalization as the mean value of each parameter I, F1, and F2 in two different time windows lasting 150 ms: the first one from 250 to 400 ms, the second one from 650 to 800 ms. The auditory representation of a vocalization is therefore a 6-dimensional vector [*I*_(1)_, *I*_(2)_, *F*1_(1)_, *F*1_(2)_, *F*2_(1)_, *F*2_(2)_]. Perceived auditory values are represented by circles on Figure 4D. Note that the agent does not have any perception of what happens before 250 ms, and that *I*_(1)_ and *I*_(2)_ can take continuous values in [0, 1] due to the averaging in a given perception time window. We will refer to the perceived “phone” of a given command for the perception occurring around the end of that command, although such an association will not be assumed in the internal sensorimotor model of the agent. Indeed, this sensorimotor system has the interesting property that the perceptions in both time windows depend on both motor commands. In the example of Figure 4, the perception for the first command, i.e., the mean auditory values between 250 and 400 ms, would not be the same if the second motor command did not cause the vocal tract opening.

#### 2.1.3. Vocalization classification

We define three types of phones, according to the value of *I* for a given command. In this description, we use common concepts like vowels or consonants to make an analogy with the human types of phones, although this analogy is limited.

- Those where *I* > 0.9, i.e., phonation occurs during almost all the 150 ms of perception around the end of the command. We call them *Vowels* (V).
- Those where *I* < 0.1, i.e., there is almost no phonation during the 150 ms of perception around the end of the command. We call them *None* (N).
- Those where 0.1 < *I* < 0.9, i.e., phonation occurs partially during the 0.15 s of perception around the end of the command. This means that the phonation level *I* has switched during that period. This can be due either to a closure or opening of the vocal tract, or to variations in the pressure and voicing parameters. We call them *Consonants* (C), although they are sometimes more comparable to a sort of prosody (when due to a variation in the phonation level).

This classification will be used as a tool for the analysis of the results in section 3, but is never known by the agent (which only has access to the values of *I*, *F*1, and *F*2).

Thus, each vocalization produced by the agent, belongs to the combination of 2 of these 3 types (because a vocalization corresponds to 2 commands), i.e., there are 3^2^ = 9 types of vocalizations: VV, VN, VC, NV, NN, NC, CV, CN, CC. An example of each type is given in the Appendix, section.

Then, we suggest to group these 9 types into 3 classes.

**Figure A1:**
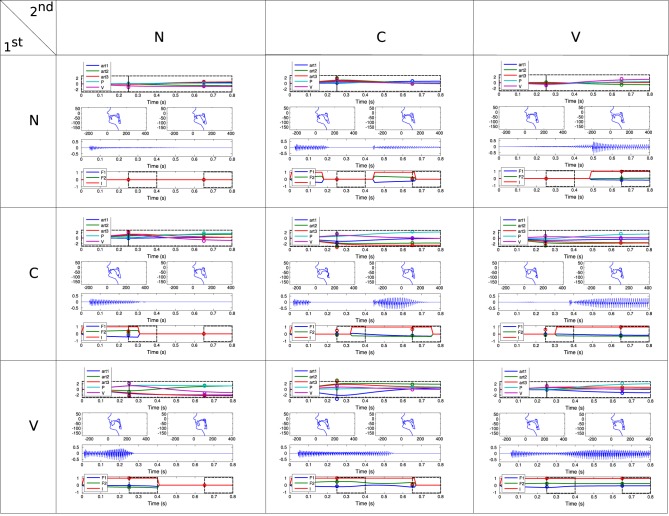
**Examples of each vocalization types**. Rows (1st) correspond to the type of the first phone and columns (2nd) to the type of the second phone of the vocalization. There are three possible phone types, as defined in section 2.1.3: the *Vowels* (V) which have a high intensity (*I* > 0.9), the *Consonants* (C) which have a low intensity (0.1 < I < 0.9) and the *None* which have almost no intensity (*I* < 0.1). For example, the plot in the second row (C) third column (V) corresponds to a CV vocalization, with the same convention as in Figure 4.

- The class *No Phonation* contains only NN: the agent has not produced an audible sound. This is due either to the fact the pressure and voicing motor variables have never been sufficiently high (not both positive, as explained in the description of the motor system) during the two 150 ms perception periods, or that the vocal tract was totally closed.
- The class *Unarticulated* contains VN, NV, CN, NC: the vocalization is not well-formed. Either the first or the second command produces a phone of type *None* (*I* < 0.1, see above).
- The class *Articulated* contains CV, VC, VV and CC: the vocalization is well-formed, in the sense that there is no *None* phone. Phonation is modulated in most cases (i.e., except in the rare case where the two commands of a VV are very similar). Note that according to the definition of *consonants*, phonation necessarily occurs in both the perception time windows (see Figure A1 in the Appendix).

It is important to note that the auditory values of these vocalization classes span subspaces of increasing complexity. Indeed, whereas various articulatory configurations belong to the *No Phonation* class, their associated auditory values are always null, inducing a 0-dimensional auditory subspace (i.e., a point). Regarding the *Unarticulated* class, the associated auditory values span a 3-dimensional subspace because at least one command produces a phone of type *None* (i.e., the corresponding auditory values are null). Finally, in the *Articulated* classes, the auditory values span the entire 6-dimensional auditory space. These properties will have important consequences for the learning of a sensorimotor model by the agent, as we will see.

### 2.2. Internal sensorimotor model

The sensorimotor internal model and the intrinsic motivation system which follow were firstly described in conference papers (Moulin-Frier and Oudeyer, 2013a,b) in a more general context where the goal was to compare various exploration strategies. In this paper, we use the active goal exploration strategy—analog to the SAGG-RIAC algorithm in Baranes and Oudeyer (2010, 2013).

During its life time, the agent iteratively updates an internal sensorimotor model by observing the auditory results of its vocal experiments. We denote motor commands *M* and sensory perceptions *S*. We call *f*: *M* → *S* the unknown function defining the physical properties of the environment (including the agent's body). When the agent produces a motor command *m* ∈ *M*, it then perceives *s* = *f*(*m*) ∈ *S*, modulo an environmental noise and sensorimotor constraints. In the sensorimotor system defined in the previous section, *M* is 18-dimensional and *S* is 6-dimensional. *f* corresponds to the transformation defined section 2.1 and illustrated Figure 4, and has a Gaussian noise with a standard deviation of 0.01. By collecting (*m*, *s*) pairs through vocal experiments, the agent learns the joint probability distribution defined over the entire sensorimotor space *SM* (therefore 24-dimensional). This distribution is encoded in a Gaussian Mixture Model (GMM) of 28 components, i.e., a weighted sum of 28 multivariate normal distributions^3^. Let us note *G*_*SM*_ this GMM. It is learnt using an online version of the Expectation-Maximization (EM) algorithm (Dempster et al., 1977) proposed by Calinon (2009) where incoming data are considered incrementally. Each update is executed once each *sm*_*step*(= 400) vocalizations are collected. *G*_*SM*_ is thus refined incrementally during the agent life, updating each time a number *sm*_*step*_ of new (*m, s*) pairs are collected. Moreover, we adapted this online version of EM to introduce a *learning rate* parameter α which decreases logarithmically from 0.1 to 0.01 over time. α allows to set the relative weight of the new learning data with respect to the old ones.

This GMM internal model is used to solve the inverse problem of inferring motor commands *m* ∈ *M* that allow the learner to reach a given auditory goal *s*_*g*_ ∈ *S*. From this sensorimotor model *G*_*SM*_, the agent can compute the distribution of the motor variables knowing a given auditory goal to reach *s*_*g*_, noted *G*_*SM*_(*M* | *s*_*g*_). This is done by Bayesian inference on the joint distribution, and results in a new GMM over the motor variables *M* (see e.g., Calinon, 2009), from which the agent can sample configurations in *M*.

**Figure 5:**
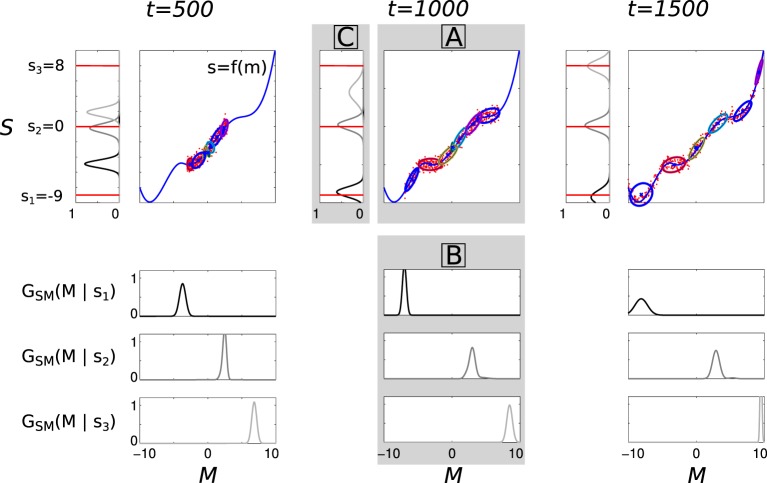
**Illustration of incremental learning and inference in the sensorimotor model in a toy 2-dimensional sensorimotor space**. The figure has three columns, corresponding to the state of a learning agent after 500, 1000, and 1500 sensorimotor experiments (*t* = 500, 1000, 1500). Each column is divided in three panels A, B, and C, as indicated in the **middle** column (boxed letters in gray panels). X-axis (*M* space) and y-axis (*S* space) of (A) are shared by (B) and (C), respectively. (A) The unknown function *s* = *f*(*m*) is represented by the blue curve. The red points are the sensorimotor experiments made at this stage (i.e., until the corresponding time index *t*): when *m* is produced, *s* = *f*(*m*) + ∈ is perceived, where ∈ is here a Gaussian noise with a standard deviation of 0.5. The ellipses represent the state of *G*_*SM*_ learned from the sensorimotor experiments, which is here a GMM with 6 components (each ellipse represents a 2D Gaussian). (B) The three vertically-aligned plots show the motor distributions *G*_*SM*_(*M* | *s*_*g*_) for 3 different goals, *s*_1_ = −9.0 **(top)**, *s*_2_ = 0.0 **(middle)**, and *s*_3_ = 8.0 **(bottom)**, in each of three columns (i.e., at the three time indexes). They are inferred from *G*_*SM*_ in (A) using Bayesian inference. (C) The probability distributions on *S* (rotated 90 degrees anti-clockwise) resulting from sampling motor configurations according to *G*_*SM*_(*M* | *s*_*g*_), to reach the three goals *s*_1_, *s*_2_, and *s*_3_, the shade of gray of each one corresponding to that used in (B): this means for example that, at a given time index *t*, producing motor commands according to the distribution *G*_*SM*_(*M* | *s*_3_) (panel B, **bottom**) will result in sensory consequences following the darker distribution in panel (C). The three considered goals *s*_1_, *s*_2_, and *s*_3_ are represented by the three horizontal red lines, which are the same in the three columns. The distributions in (C) thus reflect how the learner is able to reach one of the three considered goals using the current state of its sensorimotor model: we observe that at *t* = 500, it can only reach *s*_2_ = 0; at *t* = 1000, it can also reach *s*_1_ = −9 and at *t* = 1500 it can reach those three goals.

The whole process is illustrated Figure 5, on a toy example with mono-dimensional *M* and *S*. Given the current state of the sensorimotor model, the agent tries to achieve three goals, *s*_1_ = −9, *s*_2_ = 0, and *s*_3_ = 8, i.e., three points in *S* (how the agent is going to self-generate such goals with intrinsic motivation will be explained below). At the beginning of the life time, the model is very poor at finding a good solution because the GMM is trained with only a few data, not necessarily concentrated in the regions useful to achieve the goals. For example, at *t* = 500, the agent is only able to correctly reach *s*_2_ = 0 but is inefficient at reaching *s*_1_ = −9 and *s*_3_ = 8, as shown by the distributions over *S* in the top left corner (rotated 90 degrees anti-clockwise). Then it becomes better and better while the agent produces new vocalizations, covering a larger part of the sensorimotor space: at *t* = 1500, the agent is able to reach the three goals.

The sensorimotor system we specified in the previous section, however, involves a 24-dimensional sensorimotor space (18 articularory dimensions and 6 auditory ones). Moreover, as we have already noted, the three vocalization classes we defined (*No Phonation, Unarticulated*, and *Articulated*) span subspaces of the 6-dimensional auditory space with increasing dimensionality. Learning an inverse model using GMMs with a fixed number of Gaussians is harder, i.e., requires more sensorimotor experiments, as the spanned auditory subspace is of higher dimensionality. Although we do not provide mathematical arguments to this claim in this paper, it seems clear that learning an inverse model to produce *No Phonation* requires fewer learning data than learning an inverse model to produce various *Articulated* vocalizations, because the range of sensory effect is much larger in the second case.

### 2.3. Intrinsically motivated active exploration

In order to provide training data to the sensorimotor model we just described, the agent autonomously and adaptively decides which vocal experiments to make. The key idea is to self-generate and choose goals for which the learner predicts that experiments to reach these goals will lead to maximal competence progress.

The specific model we use in the first series of experiments (section 3.1) is a probabilistic version of the SAGG-RIAC architecture (Baranes and Oudeyer, 2010, 2013). This architecture was itself derived as a functional model (Oudeyer and Kaplan, 2007; Gottlieb et al., 2013) of theories in psychology (Berlyne, 1954; Deci and Ryan, 1985; Csikszentmihalyi, 1997; Ryan and Deci, 2000) which describe spontaneous exploration and curiosity in humans. It combines two principles: (1) goal babbling, also called goal exploration; (2) active learning driven by the maximization of empirically measured learning progress [which corresponds to the active goal strategy in Moulin-Frier and Oudeyer (2013a,b)]. In practice, the learner self-generates its own auditory goals in the sensory space *S*. One goal is here a sequence of two auditory targets encoded in a 6-dimensional vector *s*_*g*_ = [*I*_(1)_, *I*_(2)_, *F*1_(1)_, *F*1_(2)_, *F*2_(1)_, *F*2_(2)_] (see section 2.1). For each goal, it uses the current sensorimotor estimation to infer a motor program *m* ∈ *M* in order to reach that goal. Through the sensorimotor system, this produces a vocalization and the agent perceives the auditory outcome *s* ∈ *S*, hence a new (*m*, *s*) training data. Goals are selected stochastically so as to maximize the expected competence progress (i.e., the learner is interested in goals where it predicts it can improve maximally its competence to reach them at a particular moment of its development). This allows the learner to avoid spending too much time on unreachable or trivial goals, and progressively explore self-generated goals/tasks of increasing complexity. As a consequence, the learner self-explores and learns only sub-parts of the sensorimotor space that are sufficient for reachable goals: this allows to leverage the redundancy of these spaces by building dense tubes of learning data only where it is necessary for control.

We define the competence *c* associated to a particular experiment (*m*, *s*) to reach the goal *s*_*g*_ as *c* = *comp*(*s*_*g*_, s) = *e*^−‖*s*_*g*_ − *s*‖^. This measure is in [0, 1] and exponentially increases toward 1 when the Euclidean distance between the goal and the actual realization *s* = *f*(*m*) + ϵ tends to 0.

**Figure 6:**
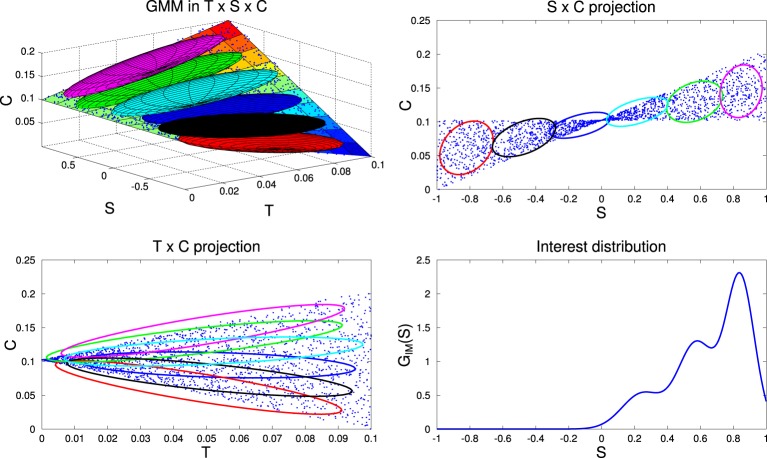
**Illustration of interest distribution computation**. **Top-Left**: the recent history of competences of the agent, corresponding to blue points in the space *T* × *S* × *C*, where *T* is the space of recent time indexes (in ℝ^+^), *S* the space of recently chosen goals *s*_*g*_ (mono-dimensional in this toy example) and *C* the space of recent competences of reaching those goals (in ℝ^+^). For the sake of the illustration, the competence variations over time are here hand-defined (surf surface) and proportional to the values in *S* (increases for positive values, decreases for negative values). We train a GMM of 6 components, *G*_*IM*_, to learn the joint distribution over *T* × *S* × *C*, represented by the six 3D ellipses. Projections of these ellipses are shown in 2D spaces *S* × *C* and *T* × *C* in the **Top-Right** and **Bottom-Left** plots. To reflect the competence progress in this dataset, we then bias the weight of each Gaussian to favor those which display a higher competence progress, that we measure as the covariance between time and competence for each Gaussian (in the example the magenta ellipse shows the higher covariance in the **Bottom-Left** plot, then the green one, the sky blue one etc). We weight the Gaussians with a negative covariance between time *T* and competence *C* (blue, black, and red ellipses) with a negligible factor, such that they do not contribute to the mixture. Using Bayesian inference in this biased GMM, we finally compute the distribution over the goal space *S*, *G*_*IM*_(*S*), thus favoring regions of *S* displaying the highest competence progress (**Bottom-Right**).

The measure of competence progress uses another GMM, *G*_*IM*_, learnt using the classical version of EM on the recent goals and their associated competences. This GMM provides an interest distribution *G*_*IM*_(*S*) used to sample goals in the auditory space *S* maximizing the competence progress in the recent sensorimotor experiments of the agent. This was firstly formalized in Moulin-Frier and Oudeyer (2013a,b). In this paper, we provide a graphical explanation of the process in Figure 6.

Following all the previous definitions, we now consider that the agent possesses the following abilities:

- Producing a complex vocalization, sequencing two motor commands interpolated in a dynamical system. It is encoded by a 18-dimensional motor configuration *m* ∈ *M*.
- Perceiving the 6-dimensional auditory consequence *s* = *f*(*m*) + ϵ ∈ *S*, computed by an articularory synthesizer. *f* is unknown to the agent.
- Iteratively learning a sensorimotor model from lots of (*m*, *s*) pairs it collects by vocalizing through time. It is encoded in a GMM *G*_*SM*_ over the 24-dimensional sensorimotor space *M* × *S*.
- Controling its vocal tract to achieve a particular goal *s*_*g*_. This is done by computing *G*_*SM*_(*M* | *s*_*g*_), the distribution over the motor space *M* knowing a goal to achieve *s*_*g*_.
- Actively choosing goals to reach in the sensory space *S* by learning an interest model *G*_*IM*_ in the recent history of experiences. By sampling in the interest distribution *G*_*IM*_(*S*), the agent favors goals in regions of *S* which maximizes the competence progress.

This agent is thus able to act at two different levels. At a high level, it chooses auditory goals to reach according to its interest model *G*_*IM*_ maximizing the competence progress. At a lower level, it attempts to reach those goals using Bayesian inference over its sensorimotor model *G*_*SM*_, and incrementally refines this latter with its new experiences. The combination of both levels results in a self-exploration algorithm (Algorithm 1).

**Algorithm 1:** **Self-exploration with active goal babbling (stochastic SAGG-RIAC architecture)**.

1:	initialise *G*_*SM*_ and *G*_*IM*_
2:	**While** true **do**
3:	*s*_*g*_ ~ *G*_*IM*_(*S*)
4:	*m* ~ *G*_*SM*_(*M* | *s*_*g*_)
5:	*s* = *f*(*m*) + ϵ
6:	*c* = *comp*(*s*_*g*_, *s*)
7:	*update*(*G*_*SM*_, (*m*, *s*))
8:	*update*(*G*_*IM*_, (*s*_*g*_, *c*))
9:	**end while**

The agent starts in line 1 with no experience in vocalizing. Both GMMs have to be initialized in order to be used. To do this, the agent acquires a first set of (*m*, *s*) pairs, by sampling in *M* around the neutral values of the articulators (see Figure 3). Regarding the pressure and voicing motor parameters, we consider that the neutral value is at −0.25, which leads to *no phonation* (recall that both these parameters have to be positive for phonation to occur, section 2.1). This models the fact that the agent does not phonate in its neutral configuration, and has at least to raise the pressure and voicing parameters to be able do do it. The agent then executes this first set of motor configurations (mostly not phonatory), observes the sensory consequences, and initializes *G*_*SM*_ with the corresponding (*m*, *s*) pairs using incremental EM. *G*_*IM*_ is initialized by setting the interest distribution *G*_*IM*_(*S*) to the distributions of the sounds it just produced with this first set of experiences. Thus, at the first iteration of the algorithm, the agent tries to achieve auditory goals corresponding to the sounds it produced during the initialization phase. Then, in the subsequent iterations, the interest distribution *G*_*IM*_(*S*) reflects the competence progress measure, and is computed as explained above.

Line 3, the agent thus selects stochastically *s*_*g*_ ∈ *S* with high interest values. Then it uses *G*_*SM*_(*M* | *s*_*g*_) to sample a vocalization *m* ∈ *M* to reach *s*_*g*_ (line 4). The execution of *m* will actually produce an auditory outcome *s* (line 5), and a competence measure to reach the goal, *c* = *comp*(*s*_*g*_, *s*), is computed (line 6). This allows it to update the sensorimotor model *G*_*SM*_ with the new (*m*, *s*) pairs (line 7). Finally, it updates the interest model *G*_*IM*_ (line 8) with the competence *c* to reach *s*_*g*_

Algorithm 1 will be run and the results analyzed in section 3.1.

### 2.4. Social (or imitation) system

In language acquisition and vocalization, the social environment plays naturally an important role. Thus we consider an active speech learner that not only can self-explore its sensorimotor space, but can also learn by imitation. In a second series of experiments (section 3.2), we extend the previous model by integrating the previous learning algorithm in the SGIM-ACTS architecture, which has been proposed in Nguyen and Oudeyer (2012).

We consider here that the learning agent can use one of two learning strategies, which it chooses adaptively:

- explore autonomously with intrinsically motivated goal babbling, as described previously,
- or explore with imitation learning. We distinguish mimicry, in which the learner copies the policies of others without an appreciation of their purpose, from emulation, where the observer witnesses someone producing an outcome, but then employs its own policy repertoire to reproduce the outcome, as formalized in Whiten (2000); Call and Carpenter (2002); Nehaniv and Dautenhahn (2007); Lopes et al. (2010). As the learner a priori can not observe the vocal tract of the demonstrator, it can only emulate the demonstrator by trying to reproduce the auditory outcome observed, by using its own means, finding its own policy to reproduce the outcome. We consider that the demonstrator (the social peer) has a finite set of auditory outcomes, and every time the learner chooses to learn by social guidance, it chooses at random an auditory outcome among the set to emulate.

The learner can monitor the competence progress resulting from using each of the strategies. This measure is used to decide which strategy is the best progress niche at a given moment: a strategy is chosen with a probability directly depending on its associated expected competence progress. Thus, competence progress is used at two hierarchical levels of active learning, forming what is called strategic learning (Lopes and Oudeyer, 2012): at the higher-level, it is used to decide when to explore autonomously, and when to imitate; at the lower-level, if self-exploration is selected, it is used to decide which goal to self-explore (as in the previous model). Since competence progress is a non-stationary measure and is continuously re-evaluated, the individual *learns* to choose both the strategy *str* ∈ {*autonomous_exploration*, *social_guidance*} and the auditory goals *s*_*g*_ ∈ *S* to target, by choosing which combination enables highest competence progress.

For the particular implementation of SGIM-ACTS of this paper, we use the same formalism and implementation as in Algorithm 1 and consider that the strategy is another choice made by the agent. This leads to Algorithm 2, where the interest model *G*_*IM*_ now learns an interest distribution as in section 2.3. The difference is that the space of interest is now the union of the strategy space {*autonomous_exploration*, *social_guidance*} and the auditory space *S*. We call *StrS* this new space *StrS* = {*autonomous_exploration*, *social_guidance*} × *S*. Hence *G*_*IM*_ is a distribution over *StrS* (Algorithm 2, line 3). If the self-exploration strategy is chosen (*str* = *autonomous_exploration*), the agent acts as in Algorithm 2. If the social guidance strategy is chosen (*str* = *social_guidance*, line 4), the learner then emulates an auditory demonstration *s*_*g*_ ∈ *S* chosen randomly among the demonstration set of adult sounds (line 5), overwriting *s*_*g*_ of line 3. It then uses its sensorimotor model *G*_*SM*_ to choose a vocalization *m* ∈ *M* to reach *s*_*g*_, by drawing according to the distribution *G*_*SM*_(*M* | *s*_*g*_) (line 7), as in the self-exploration strategy. The execution of *m* will produce an auditory outcome *s* (line 8), from which it updates its models *G*_*IM*_ and *G*_*SM*_ (lines 10 and 11).

**Algorithm 2:** **Strategic active exploration (active goal babbling and imitation with stochastic SGIM-ACTS architecture)**.

1:	Initialize *G*_*SM*_ and *G*_*IM*_
2:	**while** true **do**
3:	(*str*, *s*_*g*_) ~ *G*_*IM*_(*StrS*)
4:	**if** (*str* = *social_guidance*) **then**
5:	*s*_*g*_ ← random auditory demonstration from the ambient language
6:	**end if**
7:	*m* ~ *G*_*SM*_(*M* | *s*_*g*_)
8:	*s* = *f*(*m*) + ϵ
9:	*c* = *comp*(*s*_*g*_, *s*)
10:	*update*(*G*_*SM*_, (*m*, *s*))
11:	*update*(*G*_*IM*_, (*str*, *s*_*g*_, *c*))
12:	**end while**

Thus, this new exploration algorithm is augmented with yet another level of learning, allowing to choose between different exploration strategies. This strategy choice moreover uses the same mechanism as the choice of auditory goals, by means of the interest model *G*_*IM*_.

Algorithm 2 will be run and the results analyzed in section 3.2.

## 3. Results

The results of our experiments are presented in this section. We first run experiments where our agent learns in a pure self-exploration mode (Algorithm 1), without any social environment or sounds to imitate. In a second time, we introduce an auditory environment to study the influence of ambient language (Algorithm 2).

### 3.1. Emergence of developmental sequences in autonomous vocal exploration

**Figure 7:**
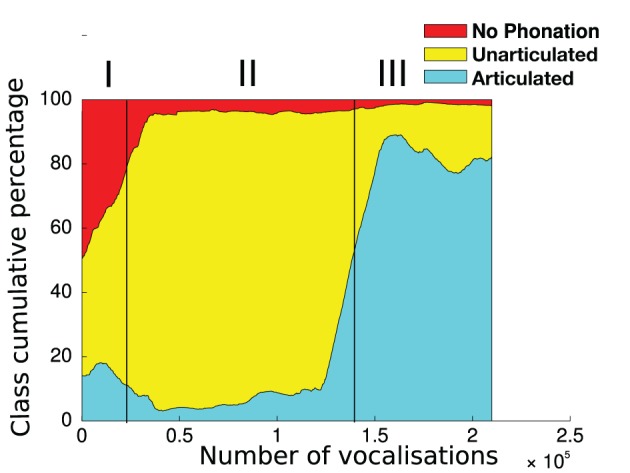
**Self-organization of vocal developmental stages**. At each time step *t* (x-axis), the percentage of each vocalization class between *t* and *t*+30,000 is plotted (y-axis), in a cumulative manner (sum to 100%). Vocalization classes are defined in section 2.1.3. Roman numerals shows three distinct developmental stages. I: mainly no phonation or unarticulated vocalizations. II: mainly unarticulated. III: mainly articulated. The boundaries between these stages are not preprogrammed and are here manually set by the authors, looking at sharp transitions between relatively homogeneous phases.

We ran 9 independent simulations of Algorithm 1 with the same parameters but different random seeds, of 240,000 vocalizations each^4^. Most of these 9 simulations display the formation of a developmental sequence, as we will see. Before describing the regularities and variations observed in this set of simulations, let us first analyse a particular one where the developmental sequence is clearly observable. Figure 7 exhibits such a simulation. We observe three clear developmental stages, i.e., three relatively homogeneous phases with rather sharp transitions. These stages are not pre-programmed, but emerge from the interaction of the vocal productions of the sensorimotor system, learning within the sensorimotor model, and the active choice of goals by intrinsically motivated active exploration. First (until ≃ 30,000 vocalizations), the agent produces mainly motor commands which results in *no phonation* or in *unarticulated* vocalizations (in the sense of the classes defined section 2.1.3). Second (until ≃ 150,000 vocalizations), phonation almost always occurs, but the vocalizations are mostly *unarticulated*. Third, it produces mainly *articulated* vocalizations.

**Figure A2:**
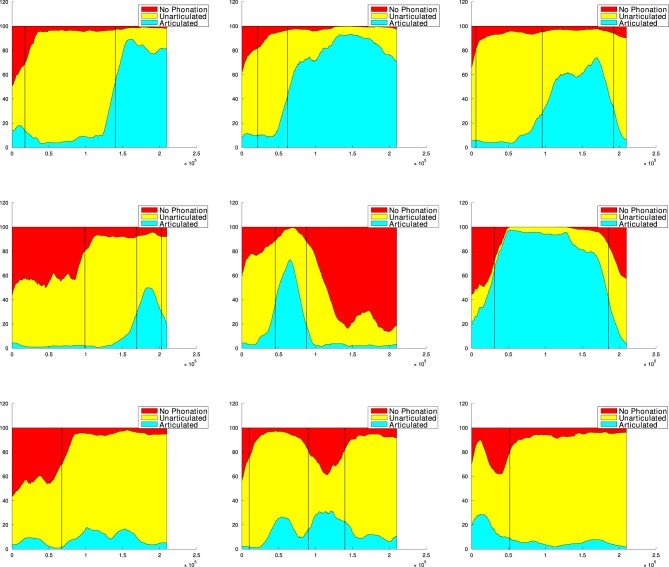
**Developmental sequences emerging from the 9 simulations for the experiment described in section 3.1**. Each subplot follows the same convention as in Figure 7. The simulations have been ordered, also in a subjective manner, from those which display a clear developmental sequence of the type *No phonation* → *Unarticulated* → *Articulated* to those less organized (from **left** to **right**, then **top** to **bottom**).

The visualization of the developmental sequence of the 9 independent simulations, provided Figure A2 in the Appendix, shows important interindividual variations whereas initial conditions are statistically similar due to initialization in line 1 of Algorithm 1. These variations can be understood through the interaction of the sensorimotor system *f*, the internal sensorimotor model *G*_*SM*_ and the interest model *G*_*IM*_, resulting in a complex dynamical system where observed developmental sequences are particular attractors (see e.g., Van Geert, 1991; Smith and Thelen, 2003). Moreover the sensorimotor and the interest models are probabilistic, thus inducing a non-negligible source of variability all along a particular simulation. Another factor is that using an online learning process on a GMM can result in a sort of forgetting, leading sometimes to the re-exploration of previously learnt parts of the sensorimotor space^5^. However, the sequence *No phonation* → *Unarticulated* → *Articulated* appears as a global tendency, as shown in Table 1. We observe that despite variations, most simulations begin with a mix of *no phonation* and *unarticulated* vocalizations, then mainly produce *unarticulated* vocalizations, and often end up with *articulated* vocalizations. An analogy can be made with human phonological systems, which are all different in the details but display strong statistical tendencies (Maddieson and Precoda, 1989; Schwartz et al., 1997; Oudeyer, 2005; Moulin-Frier et al., 2011).

**Table 1:** **Count of vocalization stages in the 9 simulations of the supplementary data**.

**Types of sounds produced**	**Stage I**	**Stage II**	**Stage III**	**Stage IV**
No phonation-unarticulated	**7**	0	2	0
Unarticulated	0	**7**	0	3
Articulated	0	2	**4**	0
Other	2	0	1	0

*The “types of sounds produced” (first column of the table) correspond to the most prominent class in a given stage, where stages are manually set, looking at sharp transitions between relatively homogeneous phases. These developmental stages are therefore subjective to a certain extent, in the sense that another observer could have set different ones (but hopefully also would observe major structural changes). “No phonation-Unarticulated” means a mix between *No phonation* and *Unarticulated* classes (as defined in section 2.1.3 in that stage). A number *x* in a cell means *this type of vocalizations (row) appears *x* times at the *n*^th^ stage of development (column) in the set of 9 simulations*. Two to four developmental stages were identified in each simulation, explaining why the “Stage I” and “Stage II” columns sum up to 9 (the total number of simulations), but not the “Stage III” and “Stage IV” columns*.

*The bold number indicates the sequence (No phonation - unarticulated) → Unarticulated → Articulated is relatively stable across simulations*.

This suggests that the agent explores its sensorimotor space by producing vocalizations of increasing complexity. The class *no phonation* is indeed the easiest to learn to produce for two reasons: the rest positions of the pressure and voicing motor parameters do not allow phonation (both around −0.25 at the initialization of the agent, line 1 of Algorithm 1); and there is no variations on the formant values, which makes the control task trivial as soon as the agent has a bit of experience. There is more to learn with *unarticulated* vocalizations, where formant values are varying in at least one part of the vocalization, and still more with *articulated* ones where they are varying in both parts (for the first and second command).

**Figure 8:**
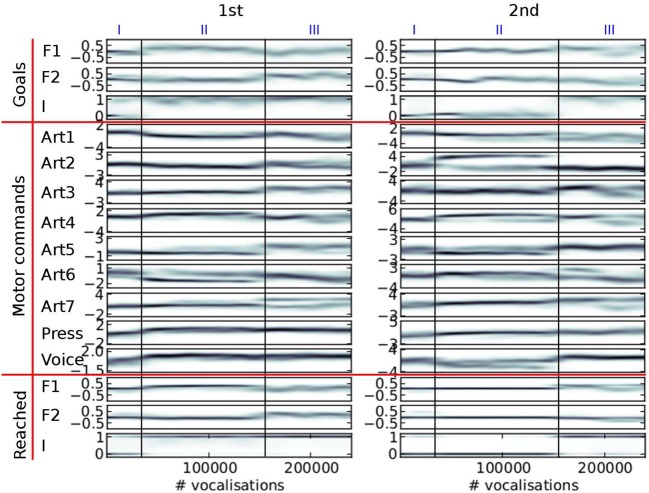
**Evolution of the distribution of auditory goals, motor commands and sounds actually produced over the life time of a vocal agent (the same agent as in Figure 7)**. The variables are in three groups (horizontal red lines): the goals chosen by the agent in line 3 of Algorithm 1 (**top** group), the motor commands it inferred to reach the goals using its inverse model in line 4 (**middle** group), and the actual perceptions resulting from the motor commands through the synthesizer in line 5 (**bottom** group). There are two columns (1st and 2nd), because of the sequential nature of vocalizations (two motor commands per vocalization). Each subplot shows the density of the values taken by each parameter (y-axis) over the life time of the agent (x-axis, in number of vocalizations since the start). It is computed using an histogram on the data (with 100 bins per axis), on which we apply a 3-bins wide Gaussian filter. The darker the color, the denser the data: e.g., the auditory parameter *I* actually reached by the second command (*I*_(2)_, last row in “Reached,” 2nd column), especially takes values around 0 (y-axis) until approximately 150,000th vocalization (x-axis), then it takes rather values around 1. The three developmental stages of Figure 7 are reported at the **top**.

Figure 8 shows what happens in the particular simulation of Figure 7 in more details.

This developmental sequence is divided into 3 stages, I, II, and III, stages being separated by vertical dark lines on Figure 8, identical on each subplot (stage boundaries are the same than in Figure 7).

In stage I, until approximately 30,000 vocalizations, the agent produces mainly *no phonation* and *unarticulated* vocalizations. We observe that the agent set goals for *I*_(1)_ either around 0, either around 1, whereas the goals for *I*_(2)_ stay around 0 (last row in “Goals”). By trying to achieve these goals, the agent progressively refines its sensorimotor model and progresses by raising the values of the pressure and voicing motor parameter in the first command (two last rows of the section “Motor commands,” 1st column). Other articulators remain around the neutral position (value 0). The agent is learning to phonate. The percentages of vocalization belonging to each vocalization class is provided Table 2.

**Table 2:** **Percentage of vocalization classes produced in stage I of the studied developmental sequence**.

**NN**	**CN**	**NC**	**VN**	**NV**	**VV**	**CV**	**VC**	**CC**
45.3%	13.4%	0.6%	18.9%	4.5%	9.9%	6.6%	0.7%	0.2%

Then, in stage II, from 30,000 to approximately 150,000 vocalizations, the agent is mainly interested in producing vocalizations which begin with a *Vowels* [*I*_(1)_ > 0.9, see the definition of phone types in section 2.1.3] and finish with a *None* [*I*_(2)_ < 0.1]. An example of such a VN vocalization can be observed in the Appendix, Figure A1 in section. During this stage, it learns to produce relatively high *F*1_(1)_ values, in particular by decreasing the *Art*_1_(1) parameter (approximately controlling the jaw height, see Figure 3). Regarding the second command, although the agent self-generates various goals for *F*1_(2)_ and *F*2_(2)_, and produces various motor commands to try to reach them, the sound produced mostly corresponds to a *None* [*I*_(2)_ = 0, and therefore *F*1_(2)_ = *F*2_(2)_ = 0]. This is due both to the negative value of the voicing parameter (last row in “Motor commands,” second column), and to the fact that the vocal tract often ends in a closed configuration due to the poor quality of the sensorimotor model in this region (because phonation occurs very rarely for the second command, leaving the agent without an adequate learning set). During this stage, the agent explores a limited part of the sensorimotor space both in time (sound only for the first command) and space (around the neutral position), until it finally manages to phonate more globally at the end of this stage. This could be correlated to the acquisition of articulated vocalizations. The percentages of vocalization belonging to each vocalization class is provided in Table 3.

**Table 3:** **Percentage of vocalization classes produced in stage II of the studied developmental sequence**.

**NN**	**CN**	**NC**	**VN**	**NV**	**VV**	**CV**	**VC**	**CC**
4.0 %	26.9 %	0.1 %	62.2 %	0.1 %	3.4 %	0.5 %	2.5 %	0.2 %

Finally, in stage III (until 150,000 to the end), phonation almost always occurs during both the perception time windows (see *I* densities, both for goals and reached values). An example of such a VV vocalization can be observed in the Appendix, Figure A1 in section. This is much harder to achieve for two reasons: firstly because there is a need to control a sequence of 2 articulators movement in order to reach two formant values in sequence [i.e., *F*1_(1)_, *F*1_(2)_, *F*2_(1)_, *F*2_(2)_] instead of one in the previous stage (the second command leading to no sound), and secondly because the position of the articulators reached for the second command also depends on the position of the articulators reached for the first one (a kind of coarticulation due to the dynamical properties of the motor system). We observe that the range of values explored in the sensorimotor space is larger than for the previous stage (both in motor and auditory spaces). The percentages of vocalizations belonging to each vocalization class is provided in Table 4.

**Table 4:** **Percentage of vocalization classes produced in stage III of the studied developmental sequence**.

**NN**	**CN**	**NC**	**VN**	**NV**	**VV**	**CV**	**VC**	**CC**
1.6 %	3.7 %	0.1 %	12.1 %	0.8 %	67.5 %	6.5 %	6.8 %	0.8 %

### 3.2. Influence of the auditory environment

**Figure 9:**
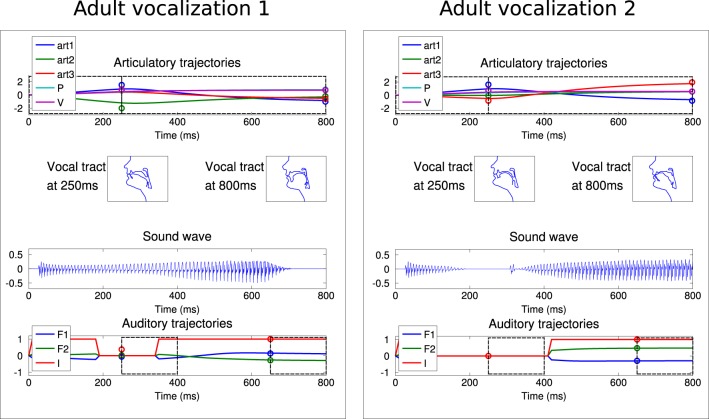
**The two vocalizations of the adult Teacher 1 used in Figure 10, with the same convention as in Figure 4**.

**Figure 10:**
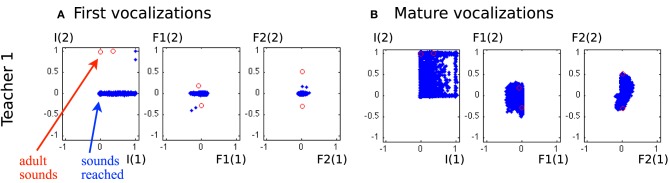
**Vocalizations of the learning agent in the early and mature stages of vocal development**. **(A)** All auditory outcomes *s* produced by the agent in its early stage of vocalization are represented by blue dots in the 6-dimensional space of the auditory outcomes. The adult sounds are represented in red circles. The actually produced auditory outcomes only cover a small area of physically possible auditory outcomes, and correspond mostly to *I*_(2)_ = 0, which represent vowel-consonant or consonant-consonant types of syllables. **(B)** The auditory outcomes produced by the infant in its mature stage of vocalization cover a much larger area of auditory outcomes and extend in particular over areas in which vocalizations of the social peer are located.

In a second set of experiments, we integrated a social environment providing a set of adult vocalizations. As explained in section 2.4, the learner has an additional choice: it can explore autonomously, or emulate the adult vocalizations. An “ambient language” is here modeled as a set of two speech sounds. To make it coherent with human language and the learning process observed in development, we chose speech-like sounds, typically vowel or consonant-vowel sounds. In terms of our sensorimotor descriptions, the adult sounds correspond to *I*1 with low values and *I*2 with high values. Figure 9 shows such vocalizations corresponding to those used by Teacher 1 in Figure 10.

Figure 10 shows a significant evolution in the agent's vocalizations. In the early stage of its development, it can only make a few sounds. Most sounds correspond to small values of *I*1(2), *F*1_(1)_, *F*1_(2)_, *F*2_(1)_, and *F*2_(2)_, as in the first developmental stage of the previous experiment (see Table 2 and Figure 8). Therefore the agent is not able to reproduce the ambient sounds of its environment. In contrast, in later periods of its development, its vocalizations cover a wider range of sounds, with notably *I*_(1)_ and *I*_(2)_ both positive, which means it now produces more articulated sounds. The development of vocalizations for a self-exploring agent in the last section showed that it progressively was able to produce articulated vocalizations, which we observed at times at the end of its development. This effect has been reinforced by the environment: with articulated vocalizations to emulate, it produces this class more regularly.

**Figure 11:**
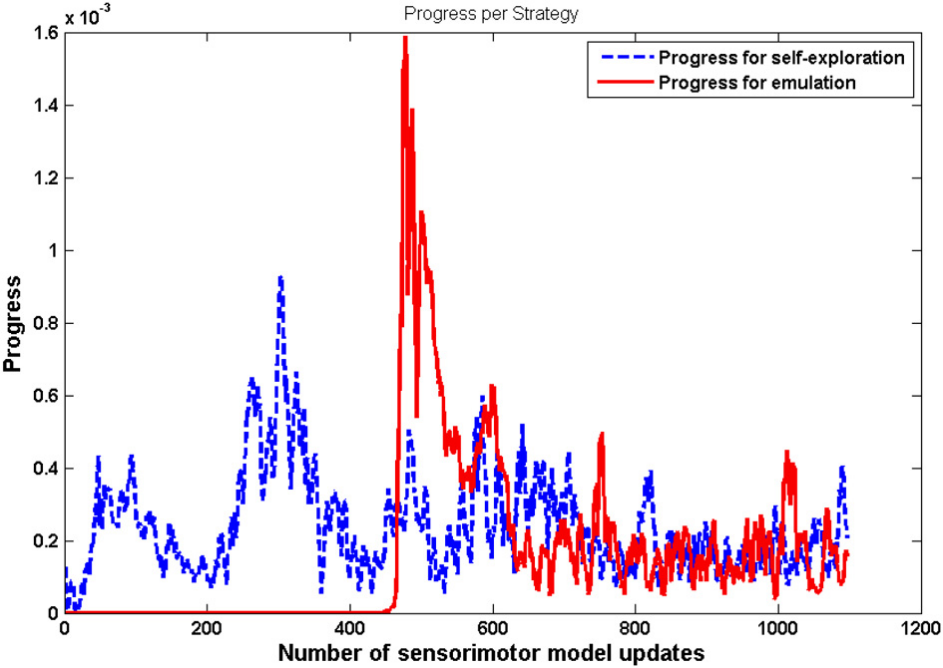
**Progress made by each strategy with respect to the number of updates of the sensorimotor model *G*_*SM*_**. These values have been smoothened over a window of 100 updates. For *t* <450, the agent makes no progress using emulation strategy. After *t* = 450, both strategies enable the agent to make progress.

**Figure 12:**
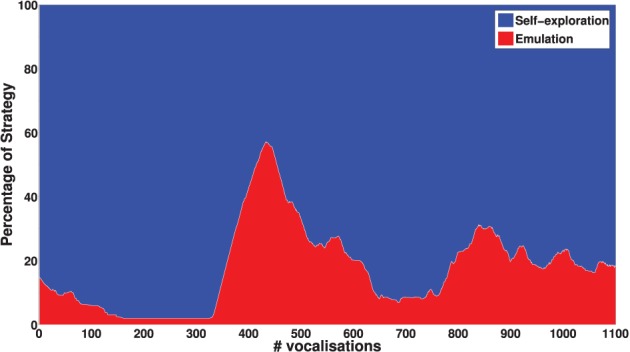
**Percentage of times each strategy is chosen with respect to the number of updates of the sensorimotor model *G*_*SM*_**. These values have been smoothened over a window of 100 updates. For *t* < 450, the agent mainly uses self-exploration strategy. When its knowledge enables it to make progress in emulation, it chooses emulation strategy until it can emulate the ambient sounds well (and its competence progress decreases).

Another important result is that mature vocalizations can now reproduce the ambient sounds of the environment: the regions of the sounds produced by the learner (blue dots) overlap the teacher's demonstrations (red circles). It seems that, during the first vocalizations, the agent cannot emulate the ambient sounds because they are too far away from its possible productions, and thus it can hardly make any progress and approach these demonstrations. Figure 11 confirms this interpretation. In the beginning, the agent makes no progress with emulation, and it is only around *t* = 450 that it makes progress with the emulation strategy. At that point, as we can see in Figure 12, it uses equally both strategies. This enables the agent to make considerable progress from *t* = 450 to *t* = 800. Indeed, once its mastery improves and the set of sounds it can produce increases, it then increasingly emulates ambient sounds. Once it manages to emulate the ambient sounds well, and thus its competence progress decreases, it uses less the emulation strategy and more the self-exploration strategy.

**Figure 13:**
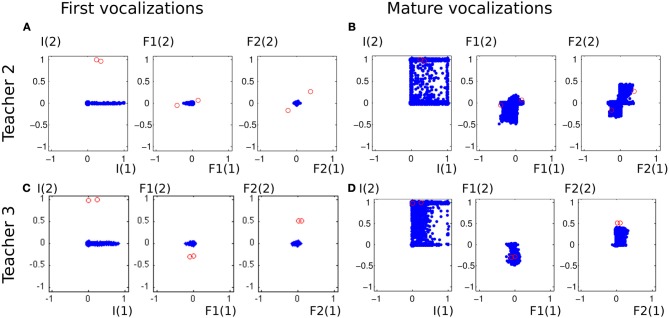
**Vocalizations of the learning agent in the early and mature stage of vocalization in two different speech environments (Teacher 2 and Teacher 3)**. **(A,C)** All auditory outcomes produced by the vocal learner in its early stage of vocal development are represented by blue dots in the 6-dimensional space of the auditory outcomes. The sounds of the environment are represented in red circles. The auditory outcomes only cover a small area, and do not depend on the speech environment. **(B,D)** The auditory outcomes produced by the infant in its mature stage of vocal development cover a larger area of auditory outcome, which depend on the speech environment.

To analyse better this emulation phenomenon and assess the influence of the ambient language, we run the same experiment with different acoustic environments. We used two other sets of speech sound demonstrations from simulated peers, and analysed the auditory productions of the agent in Figure 13. The first property that can be noted is that in the early phase of the vocal exploration (Figures 13A,C), the auditory productions of the two agents are alike, and do not depend on the speech environment. On the contrary, the mature vocalizations vary with respect to the speech environment. With Teacher 1, the productions have their values *F*2_(1)_ and *F*2_(2)_ along the axis formed by the demonstration (Figure 10A, last column). Comparatively, Teacher 2's speech sounds have different *F*1_(1)_, *F*1_(2)_, *F*2_(1)_, and *F*2_(2)_. As represented in Figure 13B, the two speech sounds now differ mainly by their *F*1_(1)_ (instead of *F*1_(2)_) and in their subspace [*F*2_(1)_, *F*2_(2)_] the speech sounds have approximately rotated from those of Teacher 1. The produced auditory outcomes of the learner look like they have changed in the same way. Whereas the reached space (blue area) seemed to be along axis *F*1_(2)_ and *F*2_(2)_ and little on *F*1_(1)_ or *F*2_(1)_ for Teacher 1, it has extended its exploration along *F*1_(2)_ and *F*2_(2)_ for Teacher 2. With Teacher 3, the demonstrations are more localized in the auditory space, with *F*1_(1)_ < 0 and *F*2_(2)_ > 0. The effect we observe in Figure 13D is that the exploration is more localized too: the explored space is more oriented toward areas where *F*1_(1)_ < 0 and *F*2_(2)_ > 0. Thus, these three examples strongly suggest a progressive influence of the auditory environment, in the sense that the first vocalizations in Figures 10, 13 are very similar, whereas we observe a clear influence of the speech environment on the produced vocalizations in later stages.

Altogether, the results of these experiments provide a computational support to the hypothesis that the progressive influence of the ambient language observed in infant vocalizations can be driven by an intrinsic motivation to maximize competence progress. At early developmental stages, attempts to imitate adult vocalizations are certainly largely unsuccessful because basic speech principles, such as phonation, are not yet mastered. In this case, focusing on simpler goals probably yields better progress niches than an imitative behavior. While they are progressively mastered, the interest in these goals decreases whereas the ability to imitate adult vocalizations increases. Imitation thus becomes a new progress niche to explore.

## 4. Discussion

Our main contribution with respect to previous computational models of speech acquisition is that we do not presuppose the existence of successive developmental stages, but rather they can emerge from an intrinsic drive to maximize the competence progress. We showed that vocal developmental stages can self-organize autonomously, from simple sensorimotor activities to more complex ones. The agent starts producing *no phonation* and *unarticulated* vocalizations, which are easy to produce because limited in the range of their auditory effects. This can be related to the first stage in infant vocal development (Figure 1), where the agent produces non speech-sounds (e.g., growls, squeals…) before learning phonation and then produces not well-articulated quasi-vowels. Later on, once the agent does not progress much in producing *unarticulated* vocalizations, it focuses on more complex vocalizations of the *articulated* class. The reason is that, due to the properties of the sensorimotor system and internal model, the mastering of complex tasks require first the mastering of simpler tasks in order to yield significant competence progress, so that these complex tasks are selected as interesting goals.

We also showed that intrinsically motivated exploration can lead to a progressive interest toward the sounds of the ambient language. Whereas the first vocalizations are mainly the result of self-exploration, they progressively lead to mastering necessary speech principles (e.g., phonation). This progressive mastering allows in turn to make significant progress in adult-speech imitation, which explains why the vocal learner starts to choose more often as targets the sound of its environment. Competence-progress based curiosity-driven exploration could thus explain a progressive influence of the ambient language on infant vocalizations.

We therefore showed that intrinsically motivated active exploration can self-organize a coherent developmental sequence, without any external clock or preset specification of this sequence. This possible role of intrinsic motivation, providing a mechanism to discover autonomously necessary developmental stages to structure the learning process, is here validated computationally. We believe that it could be of major interest for understanding the structuration of early vocal development in infants. Speech acquisition is such a complex task that intrinsic motivation could be a crucial component to make it possible in the infant's first year of life.

Our model, however, has a number of limitations. Firstly, our modeling choices of the articulatory and auditory representations, as well as the implementation of the transformation from the former to the latter, is somewhat less realistic than in some previous models: articulatory trajectories are specified using two commands per articulator with fixed durations and the auditory representation uses only three acoustic parameters (the intensity and the two first formants) averaged in fixed and relatively arbitrary perception time windows. Moreover, the fact that formant values are set to 0 whenever the intensity of the signal is null can appear quite unrealistic. Although previous models often provide more meticulous implementations of the sensorimotor system, including e.g., pitch or tactile information, what is important to us is a sensorimotor system where all vocalizations are not equally easy to learn in terms of control. Such a requirement is certainly necessary for a clear developmental sequence to emerge. Secondly, we did not treat a major issue in speech acquisition research, the so-called correspondence problem: how the child is able to relate its own vocalizations to adult vocalizations, whereas the vocal tract of the child is very different in size and geometry than the one of an adult, and therefore the spectral characteristics of the produced sounds are different. Solutions to overcome this problem have been proposed, generally based on adult feedback or reformulations associated with infant productions (Ishihara et al., 2009; Howard and Messum, 2011; Miura et al., 2012). This is outside the scope of this paper where our focus is on the self-organization of the developmental sequence in successive stages of increasing complexity. Extending our model to the interaction with real humans would definitely require to consider this issue.

Further works will consider higher-dimensional sensorimotor spaces for more realism. For example, the free software Praat (Boersma, 2012) is a powerful tool allowing to synthesize a speech signal from a trajectory in a 29-dimensional space of respiratory and oro-facial muscles. Numerous acoustic features can in turn be extracted from the synthesized sound, among which the Mel-frequency cepstral coefficients (MFCC; Davis and Mermelstein, 1980). It would also be interesting to study the effect of a vocal tract growing during the learning process, to study if our intrinsically motivated agent could re-explore only parts of the sensorimotor space which were the most affected by the vocal tract shape change. Generally, we believe that a developmental robotics approach applied to a realistic articulatory model can appropriately manage the learning process of a complex and changing mapping in high-dimensional spaces, and that observed developmental sequences can lead to interesting comparisons with infant data and predictions. Regarding the present study, such a prediction could be that a human infant should be influenced by adult sounds earlier if they were easier to produce than well-formed syllables. For example, one could imagine an experiment in which a very young infant is put in an environment where he hears external sounds that are simpler than vowels/consonants/syllables (e.g., growls) and test whether his vocalizations become influenced by external environment earlier and/or if we can measure a greater interest than in a normal speech environment.

### Conflict of interest statement

The authors declare that the research was conducted in the absence of any commercial or financial relationships that could be construed as a potential conflict of interest.

## Footnotes

^1^We have changed the gender of the subject to a male in this quotation, in order to follow the convention of the present article. Throughout this paper, we will use “he” for an infant, “she” for a caregiver (e.g., the mother) and “it” for a learning agent (the model).

^2^Available online at http://www.bu.edu/speechlab/software/diva-source-code. DIVA is a complete neurocomputational model of speech acquisition, in which we only use the synthesizer computing the articulatory-to-auditory function.

^3^We empirically chose a number of components which is a suitable trade-off between learning capacity and computational complexity.

^4^Each simulation involves several hours of computing on a desktop computer, due to the complexity of Algorithm 1, in particular in the Bayesian inference and update procedures.

^5^This is why we limited the simulations to 240,000 vocalizations each, in order to avoid this unwanted effect of forgetting. However, the fact that the system is able to adaptively re-explore sensorimotor regions that have been forgotten is an interesting feature of curiosity-driven learning.

## Appendix

### Vocalization types

Figure A1 shows the 9 types of vocalizations defined in section 2.1.3 (NN, CN, NC, VN, NV, VV, VC, CV and CC).

### Developmental sequences of 9 independent simulations

The figures of this section display the emerging developmental sequence of 9 independent simulations in pure self-exploration mode (section 3.1). At each time step *t* (x-axis), the percentage of each vocalization class during between *t* and *t*+30,000 is plotted (y-axis), in a cumulative manner. Vocalization classes are defined in section 2.1.3. For each one, we show boundaries between developmental stages. These boundaries are set manually, by looking at sharp transitions between relatively homogeneous phases. They are therefore subjective to a certain extent, in the sense that another observer could have set different ones (but hopefully also would observe major structural changes).
